# Elevated serum matrix metalloproteinase 9 (MMP-9) concentration predicts the presence of colorectal neoplasia in symptomatic patients

**DOI:** 10.1038/sj.bjc.6603958

**Published:** 2007-10-02

**Authors:** N G Hurst, D D Stocken, S Wilson, C Keh, M J O Wakelam, T Ismail

**Affiliations:** 1 Cancer Research UK Institute for Cancer Studies, University of Birmingham, Birmingham B15 2TT, UK; 2 University Hospital Birmingham NHS Trust, Edgbaston, Birmingham B15 2TH, UK; 3 Department of Primary Care and General Practice, University of Birmingham, Birmingham B15 2TT, UK

**Keywords:** colorectal, matrix metalloproteinase, screening, risk

## Abstract

Early detection of polyps or colorectal carcinoma can reduce colorectal carcinoma-associated deaths. Previous studies have demonstrated raised serum levels of matrix metalloproteinase 9 (sMMP-9) in a range of cancers. The aim of this study was to investigate the role of sMMP-9 levels in identifying colorectal neoplasia. Consenting patients donated a blood sample and were assessed by proforma-led history and physical examination. Samples were analysed for sMMP-9 concentration (enzyme-linked immuno-sorbant assay) and compared to final diagnoses. Logistic regression modelling determined independent factors associated with neoplasia. A total of 365 patients were recruited of whom 300 were analysed, including 46 normal controls. A total of 27 significant adenomas and 63 malignancies were identified. The median sMMP-9 concentration was 443ng ml^−1^ (IQR: 219–782; mean: 546). Patients with neoplasia had significantly elevated sMMP-9 levels (*P*<0.001). Logistic regression modelling identified elevated log(sMMP-9) as the most significant predictor of neoplasia (χ^2^=38.33, *P*<0.001). Other significant factors were age, sex, smoking history, abdominal pain and weight loss. The model accurately predicted neoplasia in 77.3% of cases. Sensitivity and specificity were 77.9 and 77.1%. sMMP-9 estimation can accurately stratify patient to low- or high-risk cohorts. Serum sampling is a potential means of avoiding unnecessary colonoscopy and reducing patient anxiety, iatrogenic morbidity and mortality, and cost.

Colorectal cancer remains the second commonest malignancy in England and Wales and is the target of the newest national cancer screening programme, which was officially launched in April 2006. There are more than 30 000 new cases of colorectal cancer each year in the United Kingdom and about 16 000 deaths, incurring an annual expenditure of more than £300 million in surgical, adjuvant, and palliative treatment (ONS, 2005; Macafee *et al*, 2006). Surgical advances have not improved the 5-year survival rates in the last 20 years and though an early histopathological stage at primary surgical resection correlates with increased disease-free long-term survival, 50% of patients still present with late-stage disease (Umpleby *et al*, 1984). Crude 5-year survival rates are only 35% due to the late stage of presentation of most cases (ONS, 2003). Resection of lesions in the premalignant stage permits cure without risk of occult distant metastases, therefore the detection of such early tumours is vital for a successful screening programme. Polyp excision is important in reducing the rate of progression to carcinoma in most individuals (Muto *et al*, 1975; Stryker *et al*, 1987). Excision of polyps and early tumours is associated with a good prognosis, whereas treatment of invasive carcinomas has a less favourable outcome (Dukes, 1932). Therefore, early detection of polyps or colorectal carcinoma before invasion into the surrounding mucosa represents the best hope of reducing colorectal carcinoma-associated deaths.

The majority of investigations currently employed in the assessment of patients with colorectal symptoms involve invasive procedures, exposure to significant doses of ionising radiation, or a combination of these. One or more techniques may be sequentially used in individual assessments, with increased risk accompanying multiple investigations.

While the risk of colonic perforation may be easily assumed by the very nature of flexible endoscopic approaches, it also accounts for morbidity in radiological procedures such as contrast radiology in which the sequelae of such perforations may be exaggerated by virtue of contrast media employed. Other risks include contrast reactions and delayed effects of ionising radiation exposure, which is cumulative and non-reversible. Clearly when there is commensurate benefit in the investigation, the excess risk may be partly compensated as in the staging investigation, which may follow a positive barium enema. Recent proposals suggest that quantification of the increased lifetime risk of developing a radiation-induced neoplasm should be imparted to patients prior to exposure as part of a consent process (Picano, 2004). However, it is clear that a non-invasive test, avoiding ionising radiation, would be desirable.

Non-invasive faecal occult blood testing (FOBT) relies on detection of the surrogate marker of degradation products of haemoglobin. A number of factors limit its use including the relatively poor acceptability of stool sampling and that the sought tumour must bleed – not all colorectal neoplasms bleed, or may only shed blood intermittently. Friability may be a late development arising when other symptoms are apparent. Adenomatous polyps bleed less than malignant lesions, accounting for the lower sensitivity for polyps (30% compared to *ca.* 50% for carcinomas). However data are available suggesting that use of FOBT detects colorectal lesions at an earlier stage, and that screened lesions are associated with survival benefits (Towler *et al*, 1998).

Analysis of serum samples for stratification of risk of colorectal neoplasia is untested in the clinical setting. The ideal target moiety is debatable and has centred on carcino embryonic antigen (CEA) and non-specific inflammatory markers such as C-reactive protein, erythrocyte sedimentation rate, and serum ferritin levels. While CEA may boast a degree of specificity for malignancy, it is also elevated in many diverse states including old age, obesity, smoking, inflammatory lung disease, liver disease, and other diseases of the digestive tract (Ruibal-Morell, 1992). Elevations in CEA levels are also reported in non-colorectal malignancies, including lung (74%) (Vincent and Chu, 1973; Dent *et al*, 1978), breast (57%), oesophageal, gastric, and pancreatic carcinomas. It is also more likely to be elevated in disseminated disease than in locally confined (and hence surgically curable) lesions (Beatty *et al*, 1979). Levels may decline with disease regression on treatment with chemotherapy (Bates and Longo, 1987), but up to 10% of patients with falling levels exhibit progressive disease (Kreiger *et al*, 1983).

Matrix metalloproteinases (MMPs) are zinc-dependent endopeptidases that catalyse the dissolution of the extracellular matrix classified according to domain structure into collagenases, gelatinases, stromelysins, membrane-type, and others (Kleiner and Stetler-Stevenson, 1999; Pei, 1999). The gelatinases, MMP-2 and MMP-9, have been particularly implicated in tumour invasion and metastasis formation (Poulsom *et al*, 1992; Parsons *et al*, 1998; Zeng *et al*, 1999). The primary substrate for these enzymes is type IV collagen, a major component of the basement membrane, which represents a substantial barrier for tumour cell metastasis. Most MMPs are secreted as zymogens and require activation through cleavage of a pro-domain sequence located at the N terminus of the protein.

MMPs are elevated during physiological processes, such as tissue remodelling during growth phases, pregnancy and partuition, following injury and in pathological disease states, for example chronic inflammatory processes and malignancy (Birkedal-Hansen *et al*, 1993). Their role in malignancy is highlighted by the number of cancers that have elevated MMP levels, including breast (Basset *et al*, 1990), prostatic (Pajouh *et al*, 1991), gastric (Nomura *et al*, 1995), ovarian (Campo *et al*, 1992), pancreatic (Gress *et al*, 1995; Bramhall *et al*, 1997), and colorectal disease (Hewitt *et al*, 1991; Gallegos *et al*, 1995). This led to great interest in development of MMP inhibitors as potential tumour-control agents. Unfortunately, clinical trials have failed to demonstrate successful curtailment of tumour progression (Greenwald, 1999; Steward, 1999). Nevertheless their elevation in a range of cancers prompted us to address the possibility that their levels may be elevated in the serum of patients with colorectal cancer.

## AIMS

The aims of this study were to (1) investigate the prophetic role of elevated serum matrix metalloproteinase 9 (sMMP-9) levels in identifying colorectal neoplasia within a symptomatic cohort and (2) assign and quantify relative risk of neoplasia based on serum analysis, in order to prioritise for, or even exclude from, further investigation.

## MATERIALS AND METHODS

Ethical approval was provided by South Birmingham REC and University Hospital Birmingham NHS Trust R&D office (857/04; RRK2544). All adult patients referred to a specialist colorectal clinic over a 12-month period were invited to participate. After giving informed consent, study subjects donated a sample of blood, which was encoded. All patients then were clinically assessed by proforma-led history and physical examination including rigid sigmoidoscopy. Protocol-driven invasive investigation ensued and patients were tracked to a definitive diagnosis. Serum samples were analysed for MMP-9 concentration and compared to decoded patient diagnoses. Diagnostic grouping were coined to facilitate analysis (Table 1) and a hierarchy of diagnoses was employed (data not shown) to express in simple terms the most significant pathology identified.

Patients unable or unwilling to give informed consent were excluded and referred for investigation as per clinic protocols. Patients who did not attend for subsequent investigation were excluded from the study if they did not respond to reminders generated by the imaging department (independent of the study).

### Serum preparation

Blood was harvested by single venepuncture, into plain tubes using the proprietary Vacutainer® system, prior to eliciting histories or physical examination. After clotting, serum was separated (centrifuged at 2000 *g*) and aliquoted to polyethylene Eppendorf vials for storage at −80°C until analysis.

### MMP-9 ELISA

MMP-9 ELISA kits (R&D Systems, UK) were used according to the manufacturer's instructions. Serum samples were diluted 100-fold in assay buffer. All assays were performed in duplicate on each of two enzyme-linked immuno-sorbant assay (ELISA) plates assayed 2 weeks apart, and concordance within 10% for all four wells was required. Serum MMP-9 (sMMP-9) concentrations from ‘disease-free normal control’ samples declined with advancing age (*n*=46; *R*^2^=0.027) and, therefore, age-specific predicted (expected) sMMP-9 were generated. Ratios of observed/expected values were calculated; a ratio of less or equal to 1 was considered normal, while ratios above 1 were considered pathologically elevated. A sample was denoted ‘positive’ if the observed to expected ratio exceeded 1, and a similar prospective prediction of the presence of underlying colorectal neoplasia was declared, prior to breaking the identifying sample code. The underlying definitive diagnosis was compared to the prospective prediction and accuracy recorded. Total sMMP-9 concentrations were also recorded and mapped to diagnostic group.

### Statistical analyses

To identify associations in the data, Pearson's χ^2^ test was used for categorical variables. The non-parametric Wilcoxon two-sample test (W) and Kruskal–Wallis test (KW) for more than two samples were used to compare groups of continuous measurements. Odds ratios (OR) were calculated to determine the association between exposure to possible risk factors and outcome. ORs, adjusted for all other predictive variables, were derived from logistic regression analyses. Non-neoplastic diagnoses were combined (non-neoplastic group), as were pre-malignant adenomas and colorectal carcinomas ((pre-) malignant group) in order to devise a logistic regression model to ‘predict’ patients with (pre-) malignant disease. All available factors were considered in the logistic regression analyses (gender, age, sMMP-9 level (ng ml^−1^) under a log transformation, family history, rectal bleeding, altered bowel habit, abdominal pain, weight loss, and smoking history). Logistic regression analyses using backward elimination of variables were performed using a 5% significance level to determine independent factors for prediction of those with pre-malignant or established malignant disease. Model accuracy was calculated and sensitivity, specificity, proportion of false positives, and negatives and overall percentage of correct predictions were presented. The probability cut-point was chosen to balance sensitivity and specificity.

## RESULTS

### Serum MMP-9 concentration analysis

A total of 365 patients were seen by a single clinician in the QED (Quick and Early Diagnosis) specialist colorectal clinic over a 12-month period. Onward referral for further investigation occurred in 332 cases. Informed and consenting patients donated serum for analysis. Eighteen patients declined to participate and 14 patients did not attend for their investigations. Three hundred patients with a definitive diagnosis (achieved largely by double-contrast enema) and matched serum samples were therefore enrolled in the serological arm of this study. These comprised 134 males (median (IQR) age: 62 (52–71) years) and 166 females (median (IQR) age: 64 (51–76) years). Twenty-seven significant adenomas (multiple >3, severe dysplasia, >1 cm diameter with predominant villous component) and 63 malignancies were identified in the study population. All Duke's stages were represented in the population (Stage A: 13 (20.9); B; 21 (33.9); C: 20 (32.3); D: 9 (12.9%)). Forty-six normal controls (healthy laboratory and hospital staff including retired voluntary workers) were recruited and bled (18 males, median age 52 years (inter-quartile range 46–65); 28 females, median age 62 years (inter-quartile range 51–73)).

### Observed to predicted ratios

There was a wide variation in ELISA-determined sMMP-9 concentration. Nevertheless a median sMMP-9 control value of 261ng ml^−1^ was obtained (IQR: 105–520; mean=340) in these 46 patients. Sample repeat analysis after storage (at −80°C) did not affect detected MMP9 concentrations. Several non-neoplastic conditions were diagnosed in the sample group including haemorrhoids, diverticular disease, irritable bowel syndrome, and colitis (Table 2). In each of these individual conditions the mean sMMP-9 concentration was not significantly greater than the control value. In contrast, patients with pre-malignant adenomas or colorectal carcinoma had significantly elevated sMMP-9 levels.

**Figure 1:**
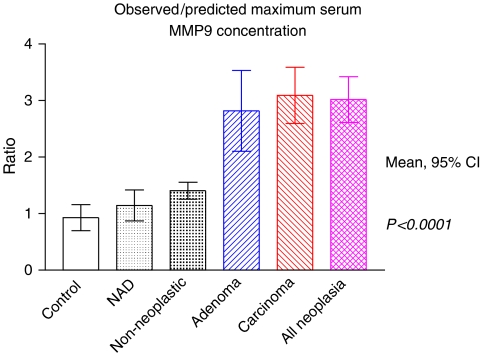
Observed to predicted maximum serum matrix metalloproteinase 9 (sMMP-9) concentrations. Highly significant differences between non-neoplastic and neoplastic conditions demonstrated.

Ratios of observed to predicted values of sMMP-9 concentration, corrected for patient age, demonstrated the ability to differentiate between non-neoplastic and neoplastic disease (Figure 1). Significant differences were demonstrable between sMMP9 concentrations in non-neoplastic and neoplastic conditions (*P*<0.0001). No statistically significant differences were identified between controls and no abnormality detected (NAD) groups (*P*=0.2794), or between NAD groups and non-neoplastic patients (*P*=0.1490).

A patient-specific prediction of the presence or absence of screenworthy pathology (see Table 1) was made based on the observed to predicted ratio of sMMP-9. This was compared to definitive diagnoses to ascertain accuracy of prediction, results of which can be seen in Table 3. The overall accuracy of this prediction was 61.0%, with a false negative rate of 1.3%; one lesion was visible in the mid-rectum giving an effective clinical false-negative rate of 1%. However, this was at the expense of an increased false-positive rate of 37.7%. Positive and negative predictive values were 44.6 and 95.8%, respectively. It is particularly noteworthy that no pre-malignant lesions were overlooked by this method. The raw cancer rate in our referred study population was 22.7%, rising to 31.4% in those with a positive serological ratio; similarly the pre-malignant adenoma rate rose from 9 to 13.2% under the same conditions. Overall (pre)-malignant lesion rates rose from 31.7 to 44.6% (a 40.7% increase). Restriction of invasive investigations to only those with positive serology could reduce imaging requirements by 32%, risking overlooking four lesions, of which one was visible in the mid-rectum, or 4.2% of screenworthy pathology. Alternatively, prioritising the patients for investigation on the basis of serological results may reduce time to detection in those with positive invasive investigations.

### Absolute serum MMP-9 concentrations

Progressing from individual patient predictions, an assessment was made of the probability of specific levels of sMMP-9, in groups with particular characteristics, indicating the presence of a screenworthy lesion. Additionally the contribution of each of several variant factors to the risk of harbouring colorectal (pre)-malignant disease was calculated.

Measured sMMP-9 concentrations were recorded against multiple factors postulated potentially relevant to modification of risk of colorectal neoplasia. Subjects were categorised as symptomatic non-neoplastic (*n*=205) including 46 normal controls, or colorectal (pre)-malignant (*n*=95). Factors considered in logistic regression analysis included sMMP-9 level (under a log transformation), age, gender, smoking history (never *vs* ever), family history (negative *vs* positive), rectal bleeding (negative *vs* positive), persistent altered bowel habit (negative *vs* positive), unexplained abdominal pain (negative *vs* positive), and unexplained weight loss (negative *vs* positive). The median sMMP-9 levels between the various non-neoplastic and control categories (see Table 1) did not significantly differ (KW χ^2^=5.69, *P*=0.58) and groups were combined as the non- (pre)-malignant group (*n*=205). sMMP-9 concentrations in the pre-malignant group (*n*=27) and the colorectal malignant group (*n*=59) were statistically similar (KW χ^2^=3.38, *P*=0.34), confirming suitability for their combination as a single group.

The risk of neoplastic disease at univariate analysis was significantly associated with increased age, male gender, increased sMMP-9, and paucity of index symptoms, other than weight loss (Table 4). Logistic regression analysis sought to identify the contribution of each variable to the risk of neoplastic disease multivariably utilising the complete data set of 300 patients in the final logistic regression model, including 95 patients with malignant or pre-malignant pathology. The final model identified sMMP-9 as the single most significant predictor of (pre)-malignant disease (χ^2^=38.33, *P*<0.001). Other significant independent predictors of neoplastic disease identified were advancing age (χ^2^=11.25, *P*<0.001), male gender (χ^2^=8.73, *P*=0.003), a negative smoking history (χ^2^=4.87, *P*=0.027), absence of abdominal pain (χ^2^=4.72, *P*=0.030), and increased weight loss (χ^2^=4.70, *P*=0.030). The other three variables (rectal bleeding, altered bowel habit, or family history) did not achieve statistical significance for inclusion, in keeping with the findings of the barium enema study previously described.

Furthermore, the risk of being in the (pre)-malignant group, attributable to a 1-unit increase in log(sMMP-9) concentration is estimated as a 5.09-fold risk (95% confidence interval (CI): 3.04, 8.51). The difference in median sMMP-9 concentrations between non-neoplastic and (pre)-malignant groups was 443ng ml^−1^. Similar calculations are possible for aging patients, who demonstrate a 4% increased risk per additional year (95% CI: 2–7%) generating a risk elevation of 66% per advancing decade of life. The model estimated increased risks of neoplastic disease for men (OR=2.52; 95% CI: 1.37, 4.66), non-smokers (OR=0.48; 95% CI: 0.25, 0.92), absence of abdominal pain (OR=0.49; 95% CI: 0.26, 0.93), and weight loss (OR=2.60; 95% CI: 1.10, 6.15) (Table 5).

**Figure 2:**
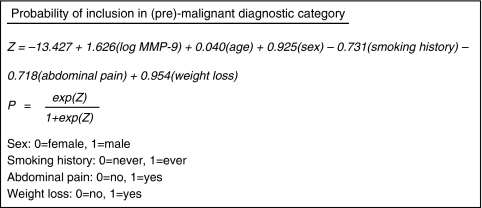
Logistic regression equations for calculation of individual risk.

A probability equation can be derived from data held in Table 5 to enable calculation of the probability of an individual patient, with defined characteristics, being included in the (pre)-malignant group (Figure 2).

Moderation of the probability threshold denoting inclusion in the (pre)-malignant group defines the accuracy of the prediction generated.

If a probability threshold to balance sensitivity and specificity is assumed, the model accurately predicts inclusion or exclusion from this group in 77.3% of patients, for this sample. Sensitivity and specificity are 77.9 and 77.1%, respectively. However, falsely negative predictions are produced in 21 individuals (11.7% of negative predictions, 7.0% of all predictions). Falsely positive predictions are derived in 47 patients (38.8% of positive predictions, 15.7% of all predictions).

Patient groups may be defined based on age, gender, smoking history, abdominal pain, weight loss, and ultimately sMMP-9 concentration to provide estimated risks of colorectal neoplasia.

In the best case scenario, a 32-year old, female smoker with abdominal pain, no weight loss, and an sMMP-9 concentration of 263, would have a calculated probability of colorectal neoplasia of 1.1%. The same woman with an sMMP-9 concentration of 1025ng ml^−1^ would have an eightfold elevation of her risk (8.9%). In the worst case scenario, a 79-year old, male, non-smoker with no abdominal pain, increased weight loss and an sMMP-9 level of 895, would have a calculated risk of colorectal neoplasia of 93.5%.

## CONCLUSIONS AND DISCUSSION

Serum MMP-9 estimation may facilitate stratifying patients referred to a specialist rapid access colorectal cancer unit into low- or high-risk cohorts, when analysed by ELISA. If our results are validated in further studies, individualised prediction of the risk of colorectal neoplasia may be inferred by comparison to local population profiles of sMMP-9 concentrations, with accuracy in excess of 76%, far exceeding that obtainable by the primary non-invasive stratification tool in current use in the United Kingdom (guaiac FOBT). While more specific methods of faecal occult blood detection are available (including immunological iFOBT, eg, Hemeselect® and Quickview®) their increased cost has, to date, precluded use in the bowel screening programme. Heightened sensitivity may be obtained by faecal DNA analysis (PreGen-Plus®) which improves detection of colorectal neoplasia from 12.9 to 51.6% compared to standard guaiac-based FOBT (Imperiale *et al*, 2004), but these techniques are expensive ($575/test at commercial rates) and their use is, at present, limited to the research setting.

Quantification of the probability of neoplasia is modifiable according to local preference. If exclusion of falsely negative test results is desirable in order to reduce the total burden of invasive imaging, sensitivity may be as high as 96.4% (rising to 99.7% in combination with clinical assessment), by altering the probability threshold calculations to favour sensitivity. If conversely, simple stratification is desirable to prioritise imaging appointments, modifying the probability threshold to 0.3 increases the positive imaging rate from 29 to 57%.

Secondary ‘sieving’ of patients with positive FOBTs returned in the screening round of the national colorectal cancer screening programme could be performed by means of ELISA quantification of sMMP-9. Negative serum samples could be excluded from confirmatory colonoscopy, significantly reducing the burden on this service. The cost savings generated could amount to a sizeable proportion of the running costs of the national FOBT screening programme. Furthermore, avoidance of unnecessary colonoscopy has the added benefit of reducing patient anxiety and potential for iatrogenic morbidity and mortality, with associated secondary cost savings accrued.

Limitations of this method of risk stratification include the introduction of human error into the assay protocol, resulting in inaccurate ELISA data with potentially damaging results. However, duplication and quality control concordance checks could help to minimise this risk and automation would reduce technical variations between assay plates.

This study was undertaken in a selected population with a high prevalence of malignancy; the usefulness of sMMP9 as a primary or secondary screening test may vary in population with different prevalence of disease. Nevertheless, this study suggests that sMMP9 may have potential application: (1) normal sMMP9 levels may be a means of identifying those at low risk of malignant disease and avoiding the need for invasive investigations and (2) raised sMMP9 levels may be a means of identifying those at increased risk of malignancy. Further research in larger, more representative populations is required to determine the usefulness of sMMP9 in stratifying both symptomatic and asymptomatic populations and determining any regional or ethnic variations in normal sMMP-9 profiles. This preliminary work has informed further ongoing work aiming to evaluate the role of sMMP9 in detecting (pre)-malignant disease in more typical populations (Ryan *et al*, 2006; Wilson *et al*, 2006).

The detection of colorectal neoplasia by this method is not infallible, but this work demonstrates that it has the potential to add to existing methodologies. It identifies that the capability exists not only for detection of established malignancy, but also for pre-malignant lesions with the same accuracy as their invasive counterparts. No other methods currently in the public domain have shown the ability to identify colorectal adenomas by non-invasive means with this degree of accuracy.

## Figures and Tables

**Table 1:** Diagnostic coding

	**Coding**
*Diagnosis*	
Normal control	1
No abnormality detected on investigation (NAD)	2
Haemorrhoids	3
Irritable bowel syndrome	4
Diverticular disease	5
Inflammatory bowel disease	6
Other anal/colorectal non-neoplastic pathology	7
Other non-colorectal non-neoplastic pathology	8
Non-premalignant polyp (metaplastic)	9
Adenomatous polyp	10
Colorectal carcinoma	11
Non-colorectal malignancy (primary diagnosis)	12
Other ‘screenworthy’ pathology (non-neoplastic)	13
	
*Diagnostic grouping*	
Normal/no screenworthy pathology	1, 2, 3, 4, 5, 6, 7, 8, 9
Normal/no detectable disease	1, 2
Benign non-screenworthy pathology	2, 3, 4, 5, 6, 7, 8, 9
Colorectal adenomatous polyp	10
Colorectal malignancy	11
Colorectal malignant and premalignant	10, 11
Screenworthy pathology	10, 11, 12, 13

Normal control: laboratory disease-free volunteers.

NAD: patients without positive findings on colorectal imaging, and includes patients with self-limiting symptoms as well as asymptomatic individuals requesting screening investigation for poor family histories in whom no lesion was identified.

Diverticular disease: patients with proven diverticulosis and symptoms compatible with the clinical manifestations of the condition, without evidence of septic complications.

Inflammatory bowel disease includes Crohn's disease, ulcerative colitis, and non-specific colitis.

Other anal/colorectal non-neoplastic pathology includes conditions such as fissure and non-specific pruritis ani.

Other non-colorectal non-neoplastic pathology includes diverse conditions such as coeliac disease, ovarian cystic disease, and vascular conditions. Metaplastic polyps were considered to have no malignant potential, while adenomatous polyps were considered potentially pre-malignant entities.

Non-colorectal malignancies included those of ovarian, gastric, haematological, and renal origin.

Screenworthy pathology (code ‘13’) includes diverticular strictures requiring surgical intervention for diagnostic discrimination from malignancy.

**Table 2:** Non-neoplastic conditions represented in symptomatic patient group

**Diagnostic category**	***n***	**%**
NAD	46	23
Haemorrhoids	25	12
Diverticular disease	85	42
Irritable bowel syndrome	7	3
Inflammatory bowel disease	14	7
Other colorectal non-neoplastic	13	6
Non-colorectal non-neoplastic	10	5
Metaplastic polyp	5	2
		
Total	205	100

Abbreviation: NAD=no abnormality detected.

**Table 3:** Comparison of prediction based on observed to predicted serum MMP-9 ratios to presence or absence of screenworthy pathology

	**Observed/predicted ratio**		
	**+ve (>1.0)**	**−ve (⩽1.0)**	
*SWP*			
Yes	91	4	95
No	113	92	205
	204	96	300

Abbreviation: SWP=screenworthy pathology.

Sensitivity: 44.6%, specificity: 95.8%, positive predictive value: 95.8%, negative predictive value: 44.9%.

**Table 4:** Univariate analysis of diagnostic category

	**Diagnostic category**			
	**(Pre)-malignant *n*=95 (%)**	**Non (pre)-malignant *n*=205 (%)**	**Odds ratio (95% CI)**	
*Sex*				
Female	43 (45%)	123 (60%)	1	^*^
Male	52 (55%)	82 (40%)	1.81 (1.11, 2.96)	
				
*Smoker*				
Never	71 (75%)	114 (56%)	1	^**^
Ever	24 (25%)	91 (44%)	0.42 (0.25, 0.73)	
				
*Family history*				
No	89 (94%)	187 (91%)	1	
Yes	6 (6%)	18 (9%)	0.70 (0.27, 1.83)	
				
*Bleeding*				
No	55 (58%)	89 (43%)	1	^*^
Yes	40 (42%)	116 (57%)	0.56 (0.34, 0.91)	
				
*Abdominal pain*				
No	68 (72%)	101 (49%)	1	^***^
Yes	27 (28%)	104 (51%)	0.39 (0.28, 0.65)	
				
*Weight loss*				
No	73 (77%)	184 (90%)	1	^**^
Yes	22 (23%)	21 (10%)	2.64 (1.37, 5.09)	
				
*Altered bowel habit*				
No	55 (58%)	92 (45%)	1	^*^
Yes	40 (42%)	113 (55%)	0.59 (0.36, 0.97)	
				

	***n***	**Median (IQR)**	**Median (IQR)**	***P* (Wilcoxon)**
*Age*	300	70 (56, 78)	61 (50, 70)	*P*<0.001
*MMP-9*	300	799 (514, 1086)	356 (160, 592)	*P*<0.001

Abbreviations: CI=confidence interval; MMP-9= metalloproteinase 9.

^***^P<0.001, ^**^P<0.01, ^*^P<0.05 when using χ^2^ analysis.

Odds ratios are given for likelihood of (pre)-malignant disease, for any given univariate discriminator.

**Table 5:** Logistic regression model for the prediction of (pre)-malignant disease

**Variable**	**Calculation**	***β***	***χ* ^2^**	***P***	**Odds ratio (95% CI)**
Intercept	—	−13.427	44.03	<0.001	—
LogMMP-9	Continuous	1.626	38.33	<0.001	5.09 (3.04, 8.51)
Age	Continuous	0.040	11.25	<0.001	1.04 (1.02, 1.07)
Sex	Female *vs* male	0.925	8.73	0.003	2.52 (1.37, 4.66)
Smoking history	Never *vs* ever	−0.731	4.87	0.027	0.48 (0.25, 0.92)
Abdominal pain	No *vs* yes	−0.718	4.72	0.030	0.49 (0.26, 0.93)
Weight loss	No *vs* yes	0.954	4.70	0.030	2.60 (1.10, 6.15)

Abbreviation: CI=confidence interval.

Adjusted *R*^2^=0.44.
